# Land cover, land use and malaria in the Amazon: a systematic literature review of studies using remotely sensed data

**DOI:** 10.1186/1475-2875-12-192

**Published:** 2013-06-08

**Authors:** Aurélia Stefani, Isabelle Dusfour, Ana Paula SA Corrêa, Manoel CB Cruz, Nadine Dessay, Allan KR Galardo, Clícia D Galardo, Romain Girod, Margarete SM Gomes, Helen Gurgel, Ana Cristina F Lima, Eduardo S Moreno, Lise Musset, Mathieu Nacher, Alana CS Soares, Bernard Carme, Emmanuel Roux

**Affiliations:** 1 EPaT Team (EA 3593), UFR de Médecine, Université des Antilles et de la Guyane, Cayenne, French Guiana; 2 STRonGer programme, Institut Pasteur de la Guyane, Cayenne, French Guiana; 3 Unité d’Entomologie Médicale, Institut Pasteur de la Guyane, Cayenne, French Guiana; 4 Instituto de Pesquisas Cientificas e Tecnológicas do Estado do Amapá, IEPA, Laboratório de Entomologia, Macapá, Amapá, Brazil; 5 Secretaria Municipal de Saúde, Oiapoque, Amapá, Brazil; 6 ESPACE-DEV, UMR228 IRD/UMII/UR/UAG, Institut de Recherche pour le Développement, Cayenne, French Guiana; 7 Laboratório Central de Saúde Pública do Amapá - LACEN-AP, Macapá, Amapá, Brazil; 8 Department of Geography, Universidade de Brasília, Brasília, Brazil; 9 Distrito Sanitário Especial Indígena - Amapá e Norte do Pará, Secretaria Especial de Saúde Indígena, Ministério da Saúde, Macapá, Brazil; 10 Laboratoire de Parasitologie, Institut Pasteur de la Guyane, Cayenne, French Guiana; 11 Centre d’Investigation Clinique – Epidémiologie Clinique Antilles-Guyane (CIC-EC INSERM CIE 802), Cayenne General Hospital, Cayenne, French Guiana; 12 Agência de Desenvolvimento do Amapá, Macapá, Amapá, Brazil

**Keywords:** Malaria, Land cover, Land use, Typology, Environmental factors, Landscape ecology, Remote sensing, Amazon

## Abstract

The nine countries sharing the Amazon forest accounted for 89% of all malaria cases reported in the Americas in 2008. Remote sensing can help identify the environmental determinants of malaria transmission and their temporo-spatial evolution. Seventeen studies characterizing land cover or land use features, and relating them to malaria in the Amazon subregion, were identified. These were reviewed in order to improve the understanding of the land cover/use class roles in malaria transmission. The indicators affecting the transmission risk were summarized in terms of temporal components, landscape fragmentation and anthropic pressure. This review helps to define a framework for future studies aiming to characterize and monitor malaria.

## Background

### Malaria patterns in Amazonia

**Figure 1:**
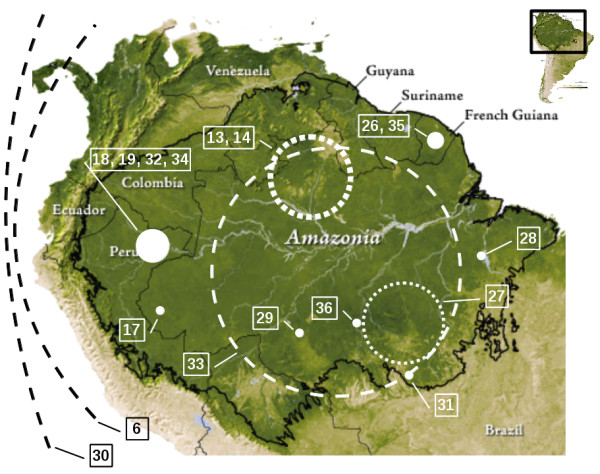
**Localization of the study areas.** Points, dotted and dashed circles and lines schematically represent, respectively, local (study areas lower than 6,000 sq km), regional (from 22,500 [27] to 225,116 sq km [14]) and large scale studies. Point size and line width are proportional to the number of studies. Circle sizes do not strictly correspond to the study area surface. Base map source: NASA (http://earthobservatory.nasa.gov/Features/AmazonEVI/).

In its broadest definition, the Amazon subregion is defined as the area covered by the humid tropical plain forest of South America that is shared by nine countries: Bolivia, Brazil, Colombia, Ecuador, France (French Guiana), Guyana, Peru, Suriname and Venezuela. The subregion covers some 7,200,000 sq km (Figure 1) and is populated by about 30 million people. The provision of health-care services to remote communities is often difficult and human mobility may limit malaria control [1].

This subregion accounted for 89% of all malaria cases in the Americas that were reported by the Pan American Health Organization (PAHO) in 2008 [2]. Among the Amazon countries, Brazil has the highest proportion of cases (56%). In 2011, Brazil and Colombia accounted for 68% of the cases in the Americas [3]. The three Guyanas (Guyana, Suriname and French Guiana) have the highest annual parasite index (API) of the Amazon subregion and, with Haiti, have the highest API of the Americas [2]. Four countries (Bolivia, Ecuador, French Guiana and Suriname) in this subregion have seen malaria incidence rates reduced by more than 75% between 2000 and 2011 but Guyana and Venezuela reported increased case numbers during this period [3].

Transmission of both *Plasmodium falciparum* and *Plasmodium vivax* occurs across the Amazon (as well as some rare *Plasmodium malariae* infections). In 2008, *P*. *vivax* accounted for 82% of the malaria burden in this subregion but with some large disparities seen between regions. *Plasmodium falciparum* was responsible for about half of the cases observed in the three Guyanas but was present in smaller proportions than *P*. *vivax* in all other Amazonian countries [2]. The proportion of cases in French Guiana and Suriname due to *P*. *falciparum* are in 2012 20% lower than they were in 2000 [3].

### The predominant role of *Anopheles darlingi*

*Anopheles darlingi* is the main malaria vector in Amazonian countries [4-6] and is the focus of most research efforts. This anopheline mosquito is widely distributed across South America and is highly anthropophilic; its biting pattern may show adaptation to human behaviour [7,8]. It is difficult to predict the occurrence of *An*. *darlingi* due to its great adaptability to different habitats and diluted presence in the environment (larva, and in some cases adults, are unlikely to be found in high densities).

Studies of *An*. *darlingi* are the most numerous but other *Anopheles* species (such as *Anopheles marajoara*) are also of interest due to their high density and entomological inoculation rates [7,9,10]. In the Amazon subregion, other anopheline species including *Anopheles braziliensis*, *Anopheles nuneztovari* and some species from the *albitarsis*, *oswaldoi* or *triannulatus* complexes may also be locally involved in malaria transmission [10-14].

### Ecological changes and “exposure risk”

The distribution of malaria is determined by climate and other geographic factors that influence the development of mosquitoes and *Plasmodium* at a given time, but it is also influenced by environmental alterations over time. Ecosystem changes resulting from natural phenomena or human interventions, on a local or global scale, can alter the ecological balance and context in which vectors and their parasites develop and transmit the disease [15]. According to Patz and Olson [16], changes in temperature patterns, due to global climate change and in variation in local land use practices, may alter malaria risk. Some authors directly relate environmental alteration to cases of malaria. Olson *et al*. [17] studied malaria in Mâncio Lima County, Brazil, in 2006. Adjusting for population, access to care and district size, a 4.3% increase in deforestation between 1997 and 2000 was associated with a 48% increase in malaria risk. Vittor *et al*. [18,19] suggested that deforestation and other human environmental alteration favour the presence of both *An*. *darlingi* larvae and adults in the Peruvian Amazon. However, Conn *et al*. [20] and Moreno *et al*. [7] suggested that human intervention could increase the presence of *An*. *marajoara* over *An*. *darling:* forest clearance and pollution may reducing the availability of larval sites for *An darlingi* and increase habitats preferred by *An*. *marajoara*.

### Land cover/use, remote sensing and malaria

Earth observation satellites permit to acquire wide ranging data concerning the continental surfaces of the Earth, with very different techniques (optical or radar imagery, radar altimetry, etc.). These data differ in their spatial, temporal, radiometric and spectral resolutions and can therefore document many environmental features at different spatial and temporal scales. The use of remote sensing (RS) to provide new insights for epidemiological studies was identified very early [21], as many diseases have been linked to environmental features. A literature review by Herbreteau *et al*. [22] in 2007 found that RS was often, and increasingly, used to study parasitic diseases (59% of studies) including malaria (16% of studies). The challenge, when studying malaria, is to identify all the natural factors (such as seasonality, rainfall, temperature, humidity, surface water and vegetation) and anthropogenic elements (such as agriculture, irrigation, deforestation, urbanization and movements of populations) of the study area, and to link them with either the incidence of disease or the presence of vectors whilst also integrating temporal and spatial variations. This would then enable the identification of risk factors from the set of possible environmental parameters.

One approach is to link malaria and the land cover (LC) and/or land use (LU) characteristics [23]. Within such a methodological framework, Ostfeld *et al*. [24] suggest that using more explicit landscape approaches to study eco-epidemiological systems could improve the understanding and prediction of the disease risk. Landscape composition (the number and types of patches) and configuration (the spatial relationships among patches) must be considered alongside the set of highly localized biotic and abiotic features. Within the framework of the study of landscape ecological functions (also referred to as landscape ecology), there are many ways to characterize the landscape, around point samples or LC/LU patches. This raises questions of objectivity, relevance and adequacy when carrying out environmental characterization. Some studies have therefore tried to standardize and evaluate the effectiveness of the characterization methods [25] or to objectify them [26].

However there has been no inventory or discussion of the LC/LU classes used for malaria studies. LC concerns the physical material observed at the earth surface (such as forests, water bodies and bare rock); LU is related to the human use of the land and integrates socio-economic and cultural functions (such as agriculture and housing).

Despite their differences, LC and LU are often mapped together and often result from remotely sensed image classifications performed by RS experts and/or botanists. Such classification procedures range from totally unsupervised approach to a full visual interpretation of the images and highly depend on the availability of the remotely sensed data, the availability of experts of the application domain, the adequacy of the data for the question addressed and the competence of the technicians, engineers and/or researchers that perform the image processing. As a result, a wide variety of LC/LU typologies and methodologies can be found in the literature. Researchers interested in malaria transmission should share their approaches to ensure that landscape characterization becomes more homogeneous and standardized. This requires a full inventory of the objectives, geographical contexts, exploited data, and LC/LU classes and their impact on the malaria transmission risk. This paper reviews the articles proposing remotely sensed LC/LU mapping for the study of malaria, to identify points of consensus and divergence, and to bring out procedural limitations. It takes an interdisciplinary point of view to formalize and unify a fragmented and sometimes implicit knowledge in the field.

## Methods

### Queries in bibliographic databases

Referenced articles using a LC/LU characterization for the study of malaria risk in the Amazon subregion were identified by performing queries in ISI Web of Knowledge^SM^ databases: Web of Science®, Medline®, Journal Citation Reports® and Current Contents Connect®. The keywords and expressions chosen to construct database queries were: *malaria*, *Anopheles darlingi*, "*land cover*" OR "*land use*", "remot* sens*", *satellite*, environment*, natural factor*, risk factor*, *deforestation*, "*South America*", Amazon*, "*Amazon basin*", America*, *tropical*, *Bolivia*, *Brazil*, *Colombia*, *Ecuador*, *French Guiana*, *Guyana*, *Peru*, Surinam*, *Venezuela*. Double quotes were used to query expressions and asterisk was used to represent any letter(s) in the query. For example, Amazon* covered the terms *Amazon*, *Amazonia*, and *Amazonian*. The queries were defined by the conjunction of two or more key words and/or expressions, resulting in the identification of between zero and 108 articles. It was not possible to identify all relevant publications using a single query (see Discussion).

### Final selection

The publications finally selected: i) are original research articles (reviews were excluded), ii) use remotely sensed LC/LU information (the study only focused on the explicit LC/LU types and ignored numeric indexes such as the NDVI), iii) are applied to malaria (malaria vectors or malaria cases) and iv) include the Amazon subregion in the study area.

## Results

Seventeen relevant articles were selected [6,13,14,17-19,26-36] according to the above-mentioned methodology (Additional file 1). Eight articles were based on epidemiological data (malaria cases or incidence), seven of them were based on entomological data (vector ecology of adults and/or larvae) and two dealt with both epidemiological and entomological data. No relevant articles were published before 2005 given the selection criteria. The frequency of publication reached a peak of four papers in 2006, and decreased in 2008.

Data from different Earth-observation satellites were used in the studies: Landsat 5 or 7 (11 publications), NOAA AVHRR (two), SPOT 5 (two), Quickbird (one), JERS-1 SAR (one) and MERIS ENVISAT (one). In one article, two different sensors were used. An increasing diversity of sensors being used was observed. In the same time, very high spatial resolution imaging (SPOT 5 and Quickbird) seems to be increasingly exploited. The majority of the study sites were in Brazil (eight publications) (Figure 1). The remaining papers concerned Peru (four articles), French Guiana (two), the whole Amazon Basin (one), the Americas (one) and worldwide (Amazon, Central Africa, Southeast Asia and Western Pacific) (one).

### Land cover/use typologies

The LC/LU types used by the authors were listed (Additional file 2). For Rosa Freitas *et al*. [13], Monteiro de Barros *et al*. [14] and Sinka *et al*. [6], who exploited LC/LU maps which had high numbers of land cover types that were not initially intended to study malaria, only the types discussed in the relevant context were listed. Comparable LC/LU types were grouped, and it was indicated if these were positively or negatively associated with malaria transmission risk. Some LC/LU types appear in several lines of the table, as they can belong to different higher level LC/LU types. For example the type *closed to open* (>*15*%) *broadleaved forest*, *regularly flooded* (*semi*-*permanently or temporarily*), *fresh or brackish water* (*Globcover channel 160*) (Sinka *et al*. [6]), belongs to both *Forest* and *Water* types.

**Figure 2:**
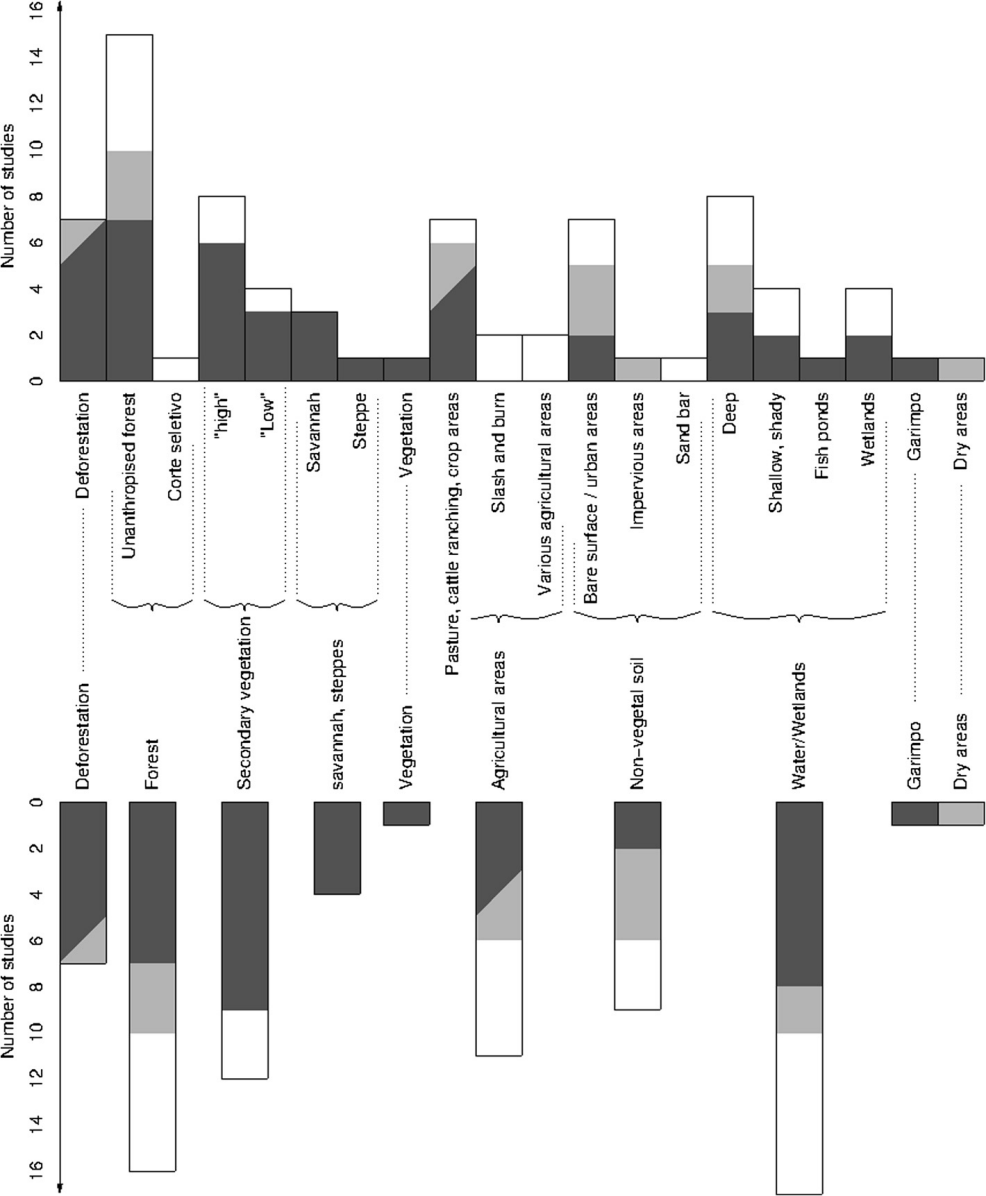
**Number of studies that assume or conclude a positive (dark gray), negative (light gray) or unknown (white) relationship between malaria and each land cover/use type found in the papers and presented in Additional file****2****.**

The number of studies that assume or conclude a positive, negative or unknown relationship between malaria and each LC/LU type are given in Figure 2. The LC/LU types correspond to those presented in Additional file 2. The positive associations between the different types of non-anthropized forests and malaria in Rosa-Freitas [13] were counted only once. Considering each forest type separately would bias the results as this study considered a much greater number of forest types than the other studies. There were no significant differences between these forest types regarding the presence of the primary malaria vectors (*An*. *darlingi* and *Anopheles albitarsis*).

## Discussion

### Paper selection

Defining simple database queries to provide all the relevant papers according to the objective was challenging; this reflects the diversity of terms used in the field. The variety of disciplinary domains interested in this topic (including epidemiology, entomology, ecology, RS and modelling) can explain such variation.

There was also potential paper selection bias due to the dominant use of the English language in the databases considered. Relevant publications in Spanish or Portuguese, underrepresented in ISI Web of Knowledge^SM^ system, could have been missed out.

The geographical criteria chosen for paper retrieval excluded some pioneering studies such as those conducted in Central America (more precisely, in Belize [37-40] and in Chiapas, Mexico [41,42]). These studies investigated the relationships between *Anopheles* abundance or larval habitats and landscape elements characterized by RS in order to predict areas at risk for malaria transmission. Studies in Central America have focused on different anopheline species from those in the Amazon subregion (*Anopheles albimanus*, *Anopheles vestitipennis*, *Anopheles punctimacula* and *Anopheles pseudopunctipennis*) with one exception [40], making it difficult to directly compare their findings with the LC/U types associated with malaria risk in this review. However, the proposed methodologies may be useful for future studies based on entomological data in the Amazon.

Some interesting and normative works concerning LC/LU mapping in the region were also ignored as this review focused solely on studies dealing with malaria. For example, the digital land cover map of South America (1 km spatial resolution) produced by Eva *et al*. in 2004 [43] could not be considered.

### Number of published papers

The availability, cost and national distribution policies of RS data (for research purposes and public health) influence the number of peer-reviewed, published papers produced. It is therefore not straightforward to interpret the distribution of published papers over time.

### Land cover/use types associated with malaria risk

The great variety of data and approaches proposed by authors justifies this review but makes it difficult to identify the underlying common aspects of the studies. Even if studies focused on a relatively homogeneous biome, local differences could result in different observations between authors. The main differences seem to originate from the disparities in objectives, study scale, data, resolution, environmental characterization and data processing approaches. Despite such variety, it was attempted to compare these papers and to identify their common points.

#### Water and wetlands

Water class (including deep water, shallow and shady water, wetlands and fishponds) was a predominant risk factor for malaria transmission because it can form vector-breeding sites. However, in two studies, the deep water class remained a protective factor against malaria as it did not allow the formation of breeding sites for mosquitoes [26,35]. For deep water bodies where water is not stagnant (streams [26,35] or water surfaces subject to wind and waves [31]), it is possible that breeding sites are located at the banks only. The configuration of banks is therefore more important than the area of the water surface. This led authors to develop specific indexes such as the length of the river banks within a given radius [26,35] or the shape index (used to differentiate lake bays that are likely to favour vector reproduction, from peninsulas which are less favourable for vector reproduction due to their exposure to wind and waves [31]).

Water type can correspond to very different habitats which may not be discriminated when using RS. For instance, optical imaging cannot identify water bodies under vegetation cover and small streams. This could explain some discrepancies between the different authors. Only one study [33] used radar images to characterize open water and wetlands. Synthetic aperture radar (SAR) data are suitable for mapping water bodies as the signal is principally sensitive surface roughness. SAR RS is able to detect flooding beneath the forest canopy and is not limited by cloud cover. The association of both optical and radar imageries can therefore be of benefit when characterizing the LC/LU in the Amazon subregion.

#### Savannah and steppes

Savannah and steppe LC/LU types were positively related to malaria in three studies and may promote the abundance of adults and/or larvae of malaria vectors. However, these types can refer to a great diversity in terms of vegetation types and densities, from herbaceous and non-ligneous vegetation (steppes, cerrado) to dense ligneous vegetation with trees that can reach 15 m (cerradão).

#### Secondary growth

A consensus emerged on the positive relation between secondary growth and increased malaria transmission risk. By studying the deforestation process, Olson *et al*. [17] found that shrub land cover (which developed five years after deforestation and was classified as secondary growth from 15 years after deforestation) had a higher abundance of *An*. *darlingi* larvae than forested land. In the reviewed studies, “secondary growth” was discussed using a wide variety of terms (including *secondary growth*, *vegetation of forest*, *vegetation in regeneration* and *fallow*). A more detailed description of such vegetation, which seems to play a major role in malaria risk transmission, should be established by authors with the help of botanists and ecologists.

#### Agriculture areas

There are apparent contradictions in study conclusions concerning agricultural activities; these seem to come from differences in LU (and not LC) types. For example, Vittor *et al*. [18,19] explain that the positive association they found between agricultural activities and malaria risk was true for slash-and-burn agriculture but did not hold in areas deforested for industrial agriculture and large-scale cattle ranches.

#### Non-vegetal soil

Only Vittor *et al*. [18,19] found a positive relationship between bare surfaces and malaria transmission risk (the presence and abundance of *An*. *darlingi*). This could be explained by the specificities of the study area. However, bare surface areas are positively correlated with areas of secondary growth, shrubs and grass/crop land (the preferred LC/LU types for *An*. *darlingi* breeding [18,19]). Positive association between bare surfaces and vector presence could therefore be a consequence of the significant association between secondary growth, shrubs, grass/crop land and the presence of vectors. Vittor *et al*. [18,19] did not attribute a direct ecological function to bare surfaces but considered them as a proxy for human activity.

#### Garimpo

Gold mining areas were positively associated with malaria in one study [27]. Their activities cause landscape changes such as the opening of the forest and the creation of puddles which are favourable for vectors. Miners can sometimes be carriers of the parasite and represent a population which are particularly vulnerable to malaria because of their living and working conditions.

#### Dense forest

Only Vittor *et al*. [18,19] found a lower malaria risk to be associated with a greater proportion of dense forest and this may be explained by the specific study area characteristics. However, like for the non-vegetal areas previously discussed, this result is not contradictory with the other study conclusions. In the studies of Vittor *et al*., high dense forest proportions were associated with relatively low proportions of secondary growth, shrubs and grass/crop land, which constitute the preferred LC/LU types for *An*. *darlingi *in breeding [18,19].

#### Deforestation

In many studies [17-19,27-30], deforested areas were associated with high malaria risk. However, this relationship must be qualified. Vittor *et al*. [18,19] contextualized their results by stating that such an association was related to a certain type of agricultural activities (see “Agriculture areas”). Other studies [27,29] confirm this differentiation and bring additional precisions by adopting a diachronic approach. They show that the association between deforestation and high malaria risk is true just after the forest clearing, but may decrease with the intensification of deforestation associated with urbanization or large cultivated areas. Within deforested areas, Barbieri *et al*. [27] and de Castro *et al*. [29] also showed that surfaces covered in low vegetation were associated with a significant risk immediately after deforestation, and a lower risk if these areas were increasing in size and were associated with urbanization.

In the Amazon, deforestation should be considered as a LC change occurring over a short time period, resulting in an abrupt opening of the dense forest. This abrupt perturbation implies vector adaptations (in distribution and density), with a transitory phase followed by stabilization if no new perturbation occurs. After six to eight years [29] after deforestation, the malaria transmission risk within the “deforested” area depends on the human activities there and their impacts on the LC; it no longer depends on the initial deforestation process. Consequently, the term “deforested area” can represent a number of realities that authors should distinguish between.

A generic model of the relationship between deforestation and malaria transmission risk emerges from the literature. It considers that: i) deforested areas can procure favourable conditions for *An*. *darlingi* breeding, ii) forest and secondary vegetation can define resting sites for adult *An*. *darlingi* mosquitoes that return to the forest after feeding, when houses are located close to the forest [8,43] and iii) that malaria transmission risk depends on the spatial distribution of LC types and, in particular, the interaction level between human populations and LC/LU types associated with breeding and resting sites.

**Figure 3:**
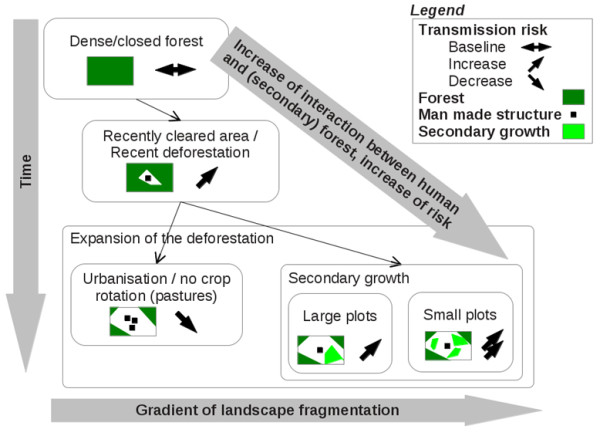
Landscape indicators that may increase or decrease malaria transmission risk as a function of time and landscape fragmentation.

This generic model is schematically represented in Figure 3. This shows the importance of distinguishing different situations by considering the LC/LU types over time in terms of both proportions and spatial distributions. It also highlights differences in agriculture practices.

### Further considerations

It should be noted that the incidence of malaria is not equivalent to the level of transmission but also depends on the level of immunity, prevention measures and treatment. The link between LC/LU characteristics and malaria data (such as incidence and prevalence) is therefore not a direct one. Concerted and effective malaria control action may initially bring down the incidence of malaria. If the action is sustainable, the transmission could be reduced due to the human population being less infective for vectors. Anthropization is often presented as a factor favouring malaria. However, when this phenomenon is present over a length of time, medical services and associated projects to prevent and fight malaria risks are often established. Human impact can therefore become a factor which reducing malaria risk without necessarily landscape modification.

Environmental features such as land cover or land use can be identified by methods other than RS. For example, some field studies have identified environmental characteristics associated with malaria risk [44,45]. However, satellite imagery can have advantages for environmental health studies as it allows a spatially complete and almost continuous characterization of the earth’s surface at increasingly high spatial and temporal resolutions. Ideally, the results of image classification should be corroborated by field validation. A landscape epidemiology approach would greatly benefit from botanist expertise in providing better characterization of landscape patches.

Malaria cases are usually geo-localized to the localities of residence of the patients or, at more local scale, to patients' home. When identifying the environmental determinants of malaria at such a very local scale, such locations may differ from those of the point of transmission, making identified relationships between environmental features and epidemiological data inaccurate. It is necessary to consider the vector or to make an assumption of suspected transmission sites in such cases. The two reviewed studies at such a very local scale [26,34] considered the links between environmental features and epidemiological data by assuming a domiciliary or peri-domiciliary transmission. This highlights difficulties in obtaining entomological data of sufficient quantity and quality, possibly due to the cost and logistic efforts required for their collection.

The choice of satellite images must not only be driven by logistical constraints; images need to be specifically selected to suit the scale of the biological phenomenon being investigated [46]. Pope *et al*. [42] proposed a hierarchical approach to determine the appropriate scale at which RS predictions of mosquito production are made.

## Conclusions

It is justified to use a landscape approach to study the eco-epidemiological system of malaria. Even though it may be extremely difficult to define a unique LC/LU typology that could be useful for the study of all malaria transmission risk issues, greater efforts should be made to enable comparison and meta-analyses of future studies. In this review, some landscape indicators that may be used as a framework for future studies aiming to characterize and monitor malaria transmission in the Amazon have been discussed. From now, greater consultation with botanists and ecologists is required to improve the characterization of LC/LU types identified with remotely sensed data and LC/LU typologies should be co-constructed with botanists, ecologists, geographers and RS experts. Deforestation is a major cause of LC change in the Amazon subregion; as this may enhance the proliferation of anopheline mosquitoes and increase malaria, further investigations based on the considerations outlined in this review should be conducted.

## Abbreviations

API: Annual parasite index; LC: Land cover; LU: Land use; NDVI: Normalized difference vegetation index; RS: Remote sensing; SAR: Synthetic aperture radar.

## Competing interests

The authors declare that they have no competing interests.

## Authors’ contributions

AS selected the publications, carried out the review and prepared the manuscript. ID participated in the data interpretation and revised the manuscript. AC, ND, AG, MC, CG, RG, MG, HG, AL, EM, LM, MN and ASS were involved in the manuscript revision. ER was responsible for overall scientific management, the interpretation of data and the preparation and revision of the manuscript. All authors read and approved the final manuscript.

## Supplementary Material

Additional file 1 **List of selected articles specifying study areas, satellites, LC/LU typologies and data used.** Papers are ordered by year of publication. Click here for file

Additional file 2 Land cover/use classes and associated indicators identified in the 17 reviewed papers. Click here for file
