# Supplementary material for: Land cover, land use and malaria in the Amazon: a systematic literature review of studies using remotely sensed data
Source: Malar J. 2013 Jun 8;12:192. doi: 10.1186/1475-2875-12-192 (PMC3684522; doi:10.1186/1475-2875-12-192)
Supplement: Additional file 1 — List of selected articles specifying study areas, satellites, LC/LU typologies and data used. Papers are ordered by year of publication. [file 1475-2875-12-192-S1.docx]

**Additional file 1 -** **List of selected articles specifying study areas, satellites, LC/LU typologies and data used**. Papers are ordered by year of publication.

| **Reference** | **Study area** | | **Satellite** | | **Land cover/use typology (Number of types: *types*, description, references)** | **Data (4)** |
| --- | --- | --- | --- | --- | --- | --- |
|  | **Localisation** | **Scale (1)** | **Sensor (2)** | **Pixel size (m) (3)** |  |  |
| Barbieri *et al* 2005 | Northern Mato Grosso State, Brazil | Regional | TM5 | 30 | 3: *urban, rural, garimpo* (from the Center of Regional Development and Planning of Brazil, Cedeplar, in collaboration with the Brazilian Health Agency FUNASA) | [E] Malaria prevalence |
| Vasconcelos *et al* 2006 | Municipality of Jacundá, Pará State,Brazil | Local | TM5 | 30 | 8: *not anthropized native vegetation, vegetation in regeneration, “corte seletivo”, agroforestry, urban area, flooded area, water, sandbar*, for years 1996 and 2001; land cover/use changes between 1996 and 2001 (deforested areas) | [E] Malaria cases, Annual parasitic index (API) |
| de Castro *et al* 2006 | Machadinho, Rondônia State, Brazil | Local | TM5 | 30 | 2: *forest and cleared land*, as a function of the time | [E] Exposure-weighted malaria illness rate (EWR) |
| Guerra *et al.* 2006* | Worldwide | Large | AVHRR |  | 1: *closed forest* (2000) and annual rates of deforestation (1990-2000), according to the Food and Agricultural Organization (FAO as part of the Forest Resources Assessment (FRA) | [E] Population at risk  [A] “Main” malaria vectors |
| Vittor *et al* 2006 | Iquitos-Nauta road, northern Peru | Local | ETM7+ | 30 | 8: forest, varillal, secondary growth, shrub, grass/crop land, bare surface, deep water, shallow water | [A] *An. darlingi* |
| Rosa-Freitas *et al* 2006* | Roraima State, Brazil | Regional | TM5, ETM7+ | 30 (1250) | 74 vegetation classes based on the nomenclature of Manual Técnico da Vegetação Brasileira the Instituto Brasileiro de Geografia e Estatística (IBGE) (IBGE, 2006);  24 land use classes based on the nomenclature of the Manual Técnico de Uso da Terra of the IBGE (IBGE, 2006) | [A] *An. darlingi, An. albitarsis, An. triannulatus, An. oswaldoi, An. nuneztovari, An. braziliensis,* *An. peryassui* |
| Zeilhofer *et al* 2007 | Manso hydropower plant reservoir, Mato Grosso State, Brazil | Local | ETM7+ | 30 | 4: *pasture/crop farming, savannah, semideciduous forest, water* (reservoir) | [A] *An. darlingi* |
| Monteiro de Barros *et al.* 2007* | Roraima State, Northern Brazilian Amazon Basin | Regional | TM5, ETM7+ | 30 (30000) | 4: *savanna, forest, mountain forest, mountain forest and savanna contact area*, according to the 74 vegetation classes of the IBGE classification (IBGE 2006) | [A] *An. darlingi, An. albitarsis, An. triannulatus, An. oswaldoi, An. nuneztovari, An. braziliensis* |

| **Reference** | **Study area** | | **Satellite** | | **Land cover/use typology (Number of types: *types*, description, references)** | **Data (4)** |
| --- | --- | --- | --- | --- | --- | --- |
|  | **Localisation** | **Scale (1)** | **Sensor (2)** | **Pixel size (m) (3)** |  |  |
| Johnson *et al* 2008 | Iquitos region, Peru | Local | TM5 | 30 | 5: *vegetation, water, shadow, manmade structures, undefined* (considered as impervious area) | [A] All collected mosquito species |
| Vittor *et al* 2009 | Iquitos-Nauta road, northern Peru | Local | ETM7+ | 30 | 8: *forest, varillal, secondary growth, shrub, grass/crop land, bare surface, deep water, shallow water* | [L] *An. darlingi* |
| Olson *et al* 2009 | Brazilian Amazon region | Large | JERS-1 SAR | 100 | 2: *open water, wetlands* | [E] Malaria incidence |
| Maheu-Giroux *et al.* 2010 | Iquitos-Nauta road, northern Peru | Local | Quickbird | 0.6 | 1: *fish ponds* | [E] Malaria incidence |
| Olson *et al* 2010* | Mancio Lima County, Acre State, Brazil | Local | TM5, ETM7+ | 30 (60) | 1: *cumulated deforested areas* from 1997 and 2000 to 2006, provided by the Programa de Cálculo do Desflorestamento da Amazônia (PRODES project) | [E] Malaria cases |
| Sinka *et al* 2010* | The Americas | Large | ENVISAT MERIS  AVHRR | 300 (5000) | 22: classes of the Globcover project (http://ionia1.esrin.esa.int/) both individually and grouped into three land cover types: *flooded, forested* and *dry areas* | [A] [L] “All” South American *Anopheles* species |
| Girod *et al* 2011 | French Guiana | Local | SPOT 5 | 10 | 9: dense forest, secondary/humid valley-floor forest, fallow, shrubs, grass land/low vegetation, bare surface, deep water, shallow/shady water, stubble-burning fields | [E] Malaria incidence  [A] *An. darlingi* |
| de Oliveira *et al* 2011 | “Vale do Amanhecer”, Mato Grosso State, Brazil | Local | TM5 | 30 | 3: *forest, agricultural area, secondary vegetation* | [E] Malaria cases |
| Stefani *et al.* 2011 | Camopi, French Guiana | Local | SPOT 5 | 10 | 9: *primary forest, secondary forest, high vegetation, medium vegetation, low vegetation, burned area, bare soil, deep water, river banks/shallow water* | [E] Malaria incidence |

* The authors did not produce the land cover/use map(s) themselves

1. Local scale corresponds to study areas lower than 6,000 sq km, for which primary epidemiological and/or entomological data were used and for which the land cover/use characterization was performed by the authors (with the exception of one of the 11 studies); Regional scale (from 22,500sq km (Barbieri *et al* 2005) to 225,116 sq km (Monteiro de Barros *et al* 2007)) corresponds to studies exploiting primary or spatially interpolated epidemiological and/or entomological data, and for which land cover/use characterization was not performed by the authors; Large scale (Americas, Brasilian Amazon region, Worldwide) corresponds to studies exploiting epidemiological and/or entomological data from state databases and existing large scale land cover/use maps (with one exception (Olson *et al* 2009)).
2. TM5: Landsat 5 TM; ETM7+: Landsat 7 ETM+; AVHRR: Advanced Very High Resolution Radiometer on National Oceanic and Atmospheric Administration (NOAA) satellites; JERS-1 SAR: Japanese Earth Resources Satellite -1 Synthetic Aperture Radar.
3. Pixel size of the primary/raw data and of the exploited data (in brackets).
4. Epidemiological data [E]; Adult [A] and/or larvae [L] vector species.
