# Supplementary material for: Land cover, land use and malaria in the Amazon: a systematic literature review of studies using remotely sensed data
Source: Malar J. 2013 Jun 8;12:192. doi: 10.1186/1475-2875-12-192 (PMC3684522; doi:10.1186/1475-2875-12-192)
Supplement: Additional file 2 — Land cover/use classes and associated indicators identified in the 17 reviewed papers. [file 1475-2875-12-192-S2.docx]

**Additional file 2 -** **Land cover/use classes and associated indicators identified in the 17 reviewed papers**

| Land cover/use type | | Description and relation with malaria risk transmission (+: positive; -: negative; ns: not significant) | | |
| --- | --- | --- | --- | --- |
| Deforestation | | Cumulative percentage of deforested areas between 10 and 5 years before (Olson 2010 [17]); Annual rates of deforestation (Guerra [30](A)); Deforested areas between 1996 and 2001 (Vasconcelos [28]); Deforested areas (Vittor 2006 and 2009 [18] [19]) | + | |
|  |  | Rural: clusters of houses in a rural area (*Sítios*) with agriculture and cattle ranching (Barbieri [27]) ; Crop area planted, pasture area, cleared area (de Castro [29]) | +/- (B) | |
| Forest | Unanthropized forest | Forest (de Oliveira [36]); Dense/primary forest (Stefani [26]); Forest (Monteiro de Barros [14](C)); Semidecidious forest (Zeilhofer [31]); Mountain forest (Monteiro de Barros [14](C)); Dense montane forest: highlands, dense montane tropical rain forest with uniform canopy (elevation >1500m), Dense submontane forest: Highlands, dense submontane tropical rain forest with emergent canopy (approximately 500-650m), Dense forest on a plateau: dense tropical rain forest on an intermediate plateau with emergent canopy at about 150-350m, Lowland forest: lowland tropical rain forest (up to 150m), Alluvial forest: lowlands, alluvial tropical rain forest, poorly drained and/or seasonally inundated, flat (up to 150m) (Rosa-Freitas [13](C)); Closed to open (>15%) broadleaved forest regularly flooded (semi-permanently or temporarily), fresh or brackish water (Globcover channel 160) (Sinka [6]) | + | |
|  |  | Closed-canopy, tall forest (Vittor 2006 and 2009 [18] [19](D)); Closed forest: natural forest (not planted by humans) constituting more than 40% of canopy cover (according to FAO) (Guerra [30](A)) | - | |
|  |  | Dense/primary forest (Girod [35]); Forested areas (Sinka [6]); Unanthropized native vegetation: all types of forest formations without human interference (Vasconcelos [28]); Varillal: naturally occurring forest growing on sandy soil that supports vegetation of lesser height and sparser canopy (Vittor 2006 and 2009 [18] [19]) | ns | |
|  | “Corte seletivo” | Selective cutting: forest areas that have undergone anthropic interference through cutting only the trees that present economic value (Vasconcelos [28]) | ns | |
| Secondary vegetation after deforestation | “High” vegetation | Secondary vegetation (De Oliveira [36]); Secondary/deteriorated forests and humid valley-floor forest (Girod [35]); Secondary growth: successive vegetation after deforestation (approximately 15 years prior) with a height of 5-15m (Vittor 2006 and 2009 [18] [19]); Fallow/high vegetation (Girod [35], Stefani [26]) | + | |
|  |  | Secondary/deteriorated forests (Stefani [26]); Vegetation in regeneration: areas with vegetation in regeneration, secondary forests or forest formations that have already suffered some type of anthropization but are not completely regenerated (Vasconcelos [28]) | ns | |
|  | “Low” vegetation | Shrubs/medium vegetation: young fallows or cultivated areas (Girod [35]); Shrubs: younger successive vegetation after deforestation (arising approximately 5 years after deforestation) with a height of 2-5m (Vittor 2006 and 2009 [18] [19]) | + | |
|  |  | Shrubs/medium vegetation: young fallows or cultivated areas (Stefani [26]) | ns | |
| Savannah | | Refers to two formations of denser scrubland or woodlands in the Cerrado biome: the Cerrado *sensu stricto* (dense, mostly mesophyllous shrubs with a grass understory) and Cerradão (woodland, mostly mesophyllous trees) (Zeilhofer [31]); Savannah (Monteiro de Barros [14](C)); Savannah: “Cerrado”, lowlands up to approximately 150m, well and poorly drained and/or seasonally inundated, flat (Rosa-Freitas [13](C)) | + | |
| **Land cover/use type** | | **Description and relation with malaria risk transmission (+: positive; -: negative; ns: not significant)** |  | |
| Steppe | | “Campos de Roraima”, highlands (up to about1000m) (Rosa-Freitas [13](C)) | + | |
| Vegetation (Johnson [32](E)) | | | + | |
| Agriculture | Pasture, cattle ranching, crop areas | Rural: Clusters of houses in a rural area (*Sítios*) with agriculture and cattle ranching (Barbieri [27]); Cleared land: planted crop areas, pasture areas, cleared areas (de Castro [29]) | +/  (B) | |
|  |  | Grass land/low vegetation (Girod [35]); Grass / crop land: cleared land that may or may not be cultivated and maintained (Vittor 2006 and 2009 [18] [19]) | + | |
|  |  | Pasture/crop farming: Natural grasslands, pastures and agricultural areas (Zeilhofer [31]) | - | |
|  |  | Grass land/low vegetation (Stefani [26]) | ns | |
|  | Slash-and-burn | Slash-and-burn agriculture areas (Girod [35], Stefani [26])(F) | ns | |
|  | Various agricultural areas | Agricultural area (de Oliveira [36]); Agroforestry: areas of bare soil which would probably be used for agriculture; livestock and agricultural areas (Vasconcelos [28]) | ns | |
| Non vegetal soil | Bare surface / Urban | Urban:s towns or villages (*povoados*) (Barbieri [27]); Manmade structure: sidewalks, roads and buildings (Johnson [32](E)); Bare soil (Stefani [26]) | - | |
|  |  | Urban area: urban patches and engineering works such as a hydroelectric plant (Vasconcelos [28]); Bare surface (Girod [35]) | ns | |
|  |  | Bare surface: compacted soil, asphalt, concrete structures, and tin roofs (Vittor 2006 and 2009 [18] [19]) | + | |
|  | Impervious areas | Manmade structures and areas classified as undefined (Johnson [32](E)) | - | |
|  | Sandbar | Islands formed by sand (Vasconcelos [28]) | ns | |
| Water | Deep water | Deep water: bodies of water at least 20 x 20 m in size, deep and with little sedimentation (Vittor 2006 and 2009 [18] [19]); Water: Dam reservoir (Zeilhofer [31](G)) | + | |
|  |  | Deep water: river streams and lakes (Girod [35], Stefani [26]) | - | |
|  |  | Open water (Olson 2009 [33](H)); Water free of vegetation, including rivers and lakes (Johnson [32]); Water (Vasconcelos [28]) | ns | |
|  | Shallow/shady water | Bodies of water at least 20 x 20 m in size that are shallow or contain large amounts of sediments. These often line the banks of deep water bodies (Vittor 2006 and 2009 [18] [19]) | + | |
|  |  | Shady rivers, parts of rivers along shady banks or wetlands composed of shallow water surfaces with sparse vegetation (Girod [35], Stefani [26]) | ns | |
|  | Fish ponds (Maheu-Giroux [34]) | | + | |
| Wetlands | | Wetland savannah, “campinarana”, lowlands (up to approximately 150m) (Rosa-Freitas [13]); Closed to open (>15%) broadleaved forest regularly flooded (semi-permanently or temporarily), fresh or brackish water (Globcover channel 160) (Sinka [6]) | + | |
|  |  | Flooded areas with aquatic vegetation (Vasconcelos [28]); Wetlands (Olson 2009 [33](H)) | ns | |
| Garimpo | | Gold mining (Barbieri [27]) | + | |
| Dry areas (Sinka [6]) | | | - | |
| 1. For Guerra, deforestation is a risk factor in South America. This is based on the assumption that there are no known closed-canopy vectors in Amazonia and that forests support a lower density and diversity of potential vectors than deforested areas.   The authors interpret this land cover over time and identify a positive association during early settlement but negative after.  This land cover type is associated with the presence of two primary vectors in the study area (*An. darlingi* and *An. albitarsis*) and potentially other secondary vectors.  These are the only studies to suggest that the amount of forest surrounding the collection sites is negatively correlated with vector occurrence.  The authors show higher diversity and density of anopheles species in rural sites. A unique non-vector species (*An. mattogrossensis*) is present in urban and peri-urban sites. *Anopheles darlingi*, the primary vector in Amazonia, is only present in rural sites, as well as other secondary vectors (*An. oswaldoi*, *An. triannulatus*).   1. Numerous confounding factors seem to affect the results related to this type (Girod [35], Stefani [26]).   Shorter distances to the reservoir border are positively related with mosquito presence. However, the authors distinguish between peninsulas and bays. Peninsulas appear to be associated with a lower probability of vector presence than bays, as the water in bays is better protected from wind and waves.  In regions with large amounts of open water or wetland, the malaria risk is negatively associated with an increase in precipitation. Mosquito habitats in wetlands, or along large rivers, may wash out or become too deep during months with high precipitation. | | | |  |
